# CBCT Evaluation of Cortical Bone Thickness in the Nasal Floor and Lateral Wall: Considerations for Implant Anchorage—A Retrospective Multicentre Study

**DOI:** 10.3390/dj13110539

**Published:** 2025-11-14

**Authors:** Fodor Romulus Calin, Bartosz Dalewski, Stefan Ihde, Marta Czuczwał, Vitomir S. Konstantinovic, Vivek Gaur, Jacek Kotuła, Łukasz Pałka

**Affiliations:** 1 Fodor Romulus Calin’s Clinic of Dentistry and Implantology, Str. Dragoş Vodă 8, 405300 Gherla, Romania; quick_dent@yahoo.com; 2 Chair and Department of Dental Prosthetics, Pomeranian Medical University, ul. Rybacka 1, 70-204 Szczecin, Poland; bartosz.dalewski@pum.edu.pl; 3 Evidence & Research Department, International Implant Foundation, Leopoldstr. 116, 80802 Munich, Germany; ihde1962@gmail.com; 4 REG-MED Dental Clinic, Rzeszowska 2, 68-200 Żary, Poland; mbienkowska94@gmail.com; 5 Clinic for Maxillofacial Surgery, School of Dentistry, University of Belgrade, Dr. Subotica 4 Street, 111000 Belgrade, Serbia; v.konstantinovic@stomf.bg.ac.rs; 6 Jaipur Dental College, Maharaj Vinayak Global University, Jaipur 302038, India; drvivekgaur@yahoo.co.in; 7 Department of Dentofacial Orthopedics and Orthodontics, Wroclaw Medical University, Krakowska 26, 50-425 Wroclaw, Poland; j_kotula@poczta.onet.pl; 8 Private Dental Practice, Rzeszowska 2, 68-200 Żary, Poland

**Keywords:** CBCT, cortical bone thickness, nasal floor, lateral wall, implant anchorage, implant stability

## Abstract

**Background/Objectives:** Primary implant stability depends on cortical bone thickness. While alveolar cortices are well studied, little is known about the nasal floor and lateral wall, which may provide alternative anchorage in atrophic maxillae. **Methods:** This retrospective, multicenter study analyzed 149 anonymized CBCT scans (83 women, 66 men; mean age 52.6 ± 13.5 years). Cortical thickness was measured at six reproducible anatomical points (A–F) defined by chosen landmarks. Measurements were taken on coronal planes aligned with implant anchorage point of interest (POI) using gray-value thresholding. Intra- and inter-observer reliability was excellent (ICC = 0.89 and 0.84). Post hoc power analysis confirmed >80% power to detect 0.15 mm differences. Non-parametric tests and mixed-effects models assessed variability and risk factors. **Results:** Thickness varied significantly by site (*p* < 0.001). The thickest cortices were at point A (median 1.36 mm, IQR 1.10–1.61) and point F (1.35 mm, 1.14–1.57), the thinnest at point B (1.15 mm, 0.96–1.32). Cortical thickness was slightly lower in men (*p* = 0.048) and decreased with age (−0.005 mm/year, *p* = 0.010). No significant associations were detected with smoking, diabetes, or thyroid disease. **Conclusions:** The anterior nasal spine and lateral wall near the sinus junction provide the greatest cortical thickness, supporting their use as potential implant anchorage sites in atrophic maxillae.

## 1. Introduction

During implant placement in the jawbones, one of the major determinants of long-term implant success is primary implant stability (PIS). Primary stability refers to the mechanical stability of a dental implant at the time of placement, which is mainly determined by the engagement between the implant surface and the surrounding bone [1,2]. Since primary stability is not a direct IS unit–based physical quantity, it is typically assessed through indirect measurements that are quantifiable in IS units. The most used parameter is insertion torque (IT), expressed in N·cm [3].

Depending on implant positioning (equicrestal or subcrestal) and design (lateral or vertical compression threads), PIS and high IT values can be achieved by compressing different types of bone tissue in various regions of the mandible and maxilla [4]. Macroscopically, two types of lamellar bone can be distinguished: trabecular and cortical. Another classification, based on density, describes these tissues as spongy (trabecular) or compact (cortical) bone. Cortical bone is mainly located near the periosteal surface, forming the outer shell of all bones, as well as lining nerve and vessel canals and natural cavities such as sinuses, orbits, and the nasal cavity [5]. It consists of osteons, each with a central vascular channel (Haversian canal) surrounded by concentric lamellae. Cortical bone is highly mineralized and exhibits lower metabolic activity and resorption rates compared to trabecular bone [6]. For these reasons, it is regarded as the most favorable bone for osseofixation of titanium implants, both in traumatology and dental implantology [7].

In recent years, most studies have focused on measuring cortical bone thickness at the alveolar crest and from the lingual/palatal and buccal aspects [8,9,10,11,12,13,14], primarily in relation to primary stability, ridge preservation, and marginal bone loss. To date, however, no studies have specifically evaluated cortical bone thickness of the nasal cavity floor, which may be used for implant anchorage, particularly in immediate loading protocols. The aim of the present study was therefore to assess cortical bone thickness at the nasal floor and its lateral wall in a multicenter population using cone beam computed tomography (CBCT). These findings are intended to provide reference data for clinicians and researchers, supporting implant treatment planning and future implant design.

## 2. Material and Methods

**Figure 1:**
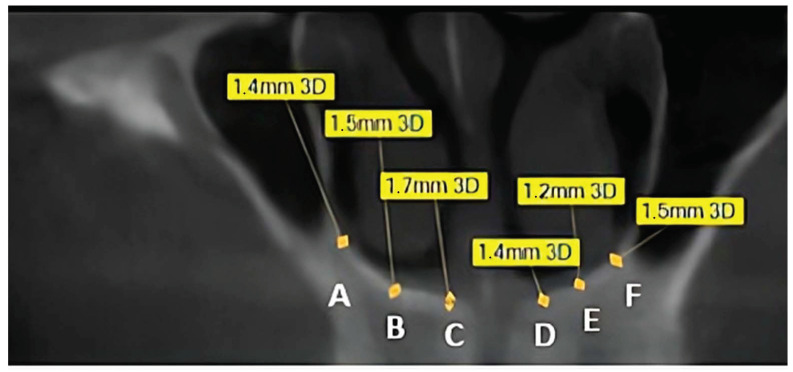
Cone beam computed tomography (CBCT) scan of the maxilla in coronal view with measurement points labeled (A–F). Yellow boxes indicate cortical bone thickness values (mm). See Table 1 for anatomical definitions of each point.

This retrospective, observational study analyzed cone beam computed tomography (CBCT) scans collected between January 2023 and December 2025 from dental clinics in Poland, Serbia, and Romania. The methodology followed the same protocol as in previous studies conducted by the research group. All CBCT scans were acquired using the Pax-3D Smart Tomograph (Vatech, Hwaseong, Republic of Korea) with a voxel size of 0.2 mm, exposure settings of 8.4 mA and 94 kV, and a scanning time of 18 s. The cylindrical field of view measured 100 × 80 mm. Images were reconstructed with a slice thickness of 0.2 mm at 0.2 mm intervals. The dataset comprised 149 CBCT scans of the anterior maxilla, retrospectively selected from patient archives. Only cases with complete visualization of the nasal bone, particularly the floor of the nasal cavity and its lateral walls, were included. All scans were anonymized prior to analysis, including demographic data and systemic risk factor information (smoking, diabetes, thyroid disorders), ensuring no patient identifiers were available. Since images were originally acquired for routine clinical purposes, patient consent was not required. To minimize selection bias, eligible scans were randomly selected using a computer-generated randomization algorithm. At no point did the researchers have access to identifiable patient information. Cortical bone thickness was measured independently by two experienced implantologists using specialized imaging software (Ez3D-i, version 1.0.6.0.1, Vatech, Republic of Korea) on coronal radiological planes (Figure 1). To assess intra- and inter-observer reliability, a subset of scans was re-evaluated after several weeks. Two additional independent researchers reviewed the measurements, and discrepancies were resolved by consensus. Discrepancies between observers were reviewed for all measurements, not only the subset reevaluated, and resolved by consensus. A post hoc power analysis indicated that the sample size (*n* = 149) provided >80% power to detect cortical thickness differences of 0.15 mm between anatomical sites at α = 0.05. The final dataset included 149 patients (83 women and 66 men) aged 14–80 years (mean: 52.6 ± 13.5 years). In each case, cortical bone thickness was measured at six predefined anatomical points (A–F) (Figure 1). Data on risk factors were also collected: 18.1% of patients were smokers, 10.1% had diabetes, and 8.1% had thyroid disorders.

**Table 1:** Measurement landmarks (Points A–F, Figure 1). Six standardized (three corresponding) anatomical points were selected along the floor of the nasal cavity and its lateral walls.

Points A and F	Base point of the medial wall of the maxillary sinus and nasal cavity.
Points B and E	Middle point of interest POI area.
Points C and D	Point 2mm from naso-palatine canal wall.

Slice selection. Coronal slices were selected at the line perpendicular to the alveolar crest between canine and premolar point in dentate patients and at the base of distal wall of naso-palatine canal in edentulous one. This ensured alignment with clinically realistic boundaries of implant placement. The contralateral point of interest (POI) area on both sides was marked with two points and divided in half at the middle creating third measure point. Measurements were performed on coronal planes at 0.2 mm slice thickness, ensuring reproducibility.

Cortical delineation protocol. Cortical boundaries were defined using the gray-value threshold function of the Ez3D-i software. Only the hyperdense cortical margin was included; trabecular bone was excluded by selecting the densest line at the inner/outer cortical edge. Measurements were taken perpendicular to the cortical surface.

Reliability assessment. To ensure reproducibility, intra- and inter-observer intraclass correlation coefficients (ICCs) were calculated from a random subset of 30 scans. Agreement was excellent (ICC intra-observer = 0.89; ICC inter-observer = 0.84).

Statistical analyses were performed to evaluate differences in cortical bone thickness between measurement points and to identify potential influencing factors. The Shapiro–Wilk test was used to assess normality at each measurement point. Since most distributions deviated from normality, differences between dependent measurements were analyzed using the Friedman test for repeated measures, followed by pairwise comparisons with the Wilcoxon signed-rank test and Bonferroni correction. To examine the effects of individual factors (sex, smoking status, diabetes, thyroid disease, and age) on cortical bone thickness, a linear mixed-effects model was applied with patients as random effects and the listed factors as fixed effects. Between-group comparisons were additionally performed using Student’s *t*-tests. Effect sizes were calculated using Cohen’s d. The relationship between cortical bone thickness and age was further evaluated using Pearson’s and Spearman’s correlation coefficients, as well as multivariate linear regression.

## 3. Results

### 3.1. Differences in Bone Thickness Between Measured Points

**Figure 2:**
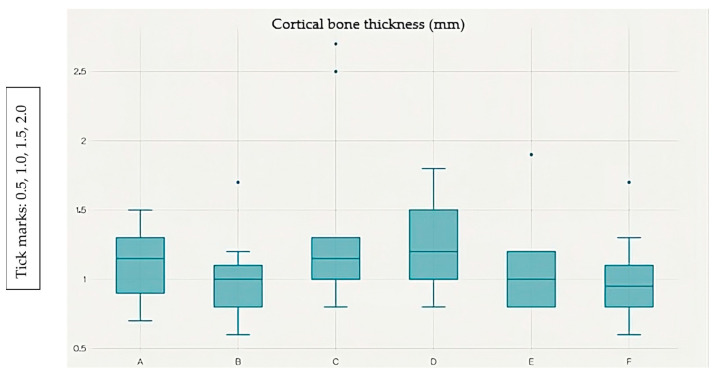
Box plot showing the distribution of cortical bone thickness (mm) at measurement points A–F. The median, interquartile range (IQR), and outliers are displayed.

The Shapiro–Wilk test indicated that most measurement points (5 out of 6) deviated from a normal distribution (*p* < 0.05). Therefore, non-parametric tests were applied. The Friedman test revealed significant differences in cortical bone thickness across the six anatomical points (χ^2^ = 43.77, df = 5, *p* < 0.001). These findings confirm that cortical thickness varies significantly depending on the measurement location (Figure 2).

Points A and F exhibit the greatest cortical thickness, whereas point B shows the lowest values. Post hoc analysis with the Wilcoxon signed-rank test and Bonferroni correction (α = 0.0033) identified six significant pairwise differences out of 15 possible comparisons: A vs. B (*p* < 0.001), A vs. C (*p* = 0.0018), B vs. D (*p* = 0.0002), B vs. E (*p* < 0.001), B vs. F (*p* < 0.001), and C vs. F (*p* = 0.0019).

The cortical bone thickness data were not normally distributed; therefore, they are reported as median values with corresponding interquartile ranges (IQR). Thickness values were: A = 1.36 (1.10–1.61) mm; F = 1.35 (1.14–1.57) mm; E = 1.34 (1.02–1.71) mm; D = 1.25 (1.08–1.44) mm; C = 1.23 (1.04–1.44) mm; B = 1.15 (0.96–1.32) mm.

These results indicate that cortical bone was thickest at points A and F, and thinnest at point B, with statistically significant variations observed between specific measurement sites.

### 3.2. Influence of Risk Factors on Cortical Bone Thickness

**Figure 3:**
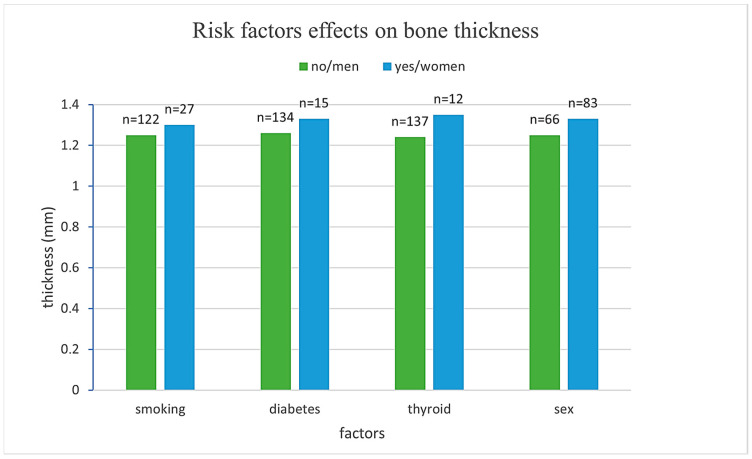
Comparison of mean bone thickness across different risk factor groups.

A linear mixed-effects model with random effects for patients and fixed effects for the analyzed variables was applied. The model results are summarized in Figure 3.

Men exhibited, on average, 0.098 mm thinner cortical bone compared to women (*β* = −0.098, SE = 0.050, *p* = 0.048), representing a statistically significant difference. Smoking, diabetes, and thyroid disease were not associated with significant differences in cortical thickness (*p* = 0.700, 0.152, and 0.546, respectively). Age demonstrated a significant negative association with cortical bone thickness, with each additional year of life corresponding to a decrease of 0.005 mm (*β* = −0.005, SE = 0.002, *p* = 0.010) as presented in Table 2.

These findings suggest that sex and age are significant predictors of cortical bone thickness, while smoking, diabetes, and thyroid disease do not exert a measurable influence in this sample.

Reliability of measurements. The intra-observer ICC was 0.89 (95% CI: 0.83–0.94), and the inter-observer ICC was 0.84 (95% CI: 0.76–0.91), confirming excellent agreement in cortical thickness assessment.

### 3.3. Relationship Between Cortical Bone Thickness and Age

**Figure 4:**
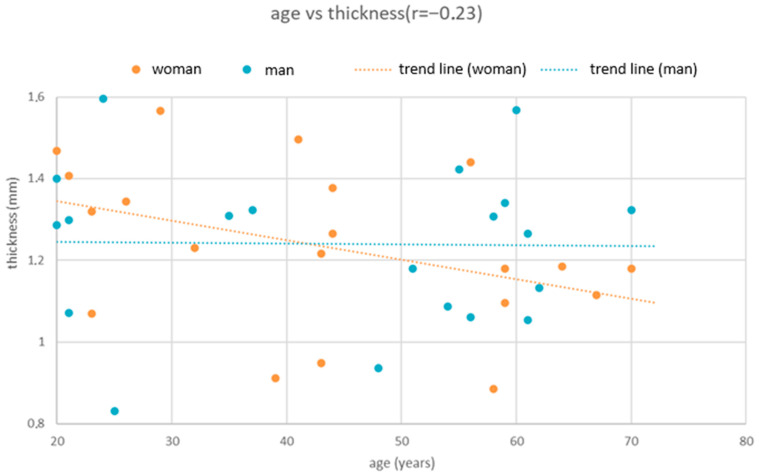
Scatter plot illustrating the relationship between age and cortical bone thickness. A weak but statistically significant negative correlation was observed (Spearman’s ρ = −0.194, *p* = 0.018; Pearson’s r = −0.175, *p* = 0.033). The fitted regression line (thickness = 1.492 − 0.004 × Age, R^2^ = 0.031) indicates a decrease in cortical bone thickness of approximately 0.004 mm per year.

Correlation analysis demonstrated a weak but statistically significant negative relationship between age and cortical bone thickness (Figure 4). Spearman’s correlation yielded ρ = −0.194 (*p* = 0.018), while Pearson’s correlation showed r = −0.175 (*p* = 0.033). Both measures confirmed that increasing age is associated with reduced cortical thickness.

A simple linear regression model further supported this association (thickness = 1.492 − 0.004 × Age). The model explained 3.1% of the variance in bone thickness (R^2^ = 0.031). According to the regression coefficient, each additional year of life corresponds to a decrease in cortical bone thickness of approximately 0.004 mm.

### 3.4. Variability Characteristics

The mixed-effects model revealed that a considerable proportion of the variance in cortical bone thickness originated from within-patient differences across measurement points. The variance structure is presented in Table 3.

The intraclass correlation coefficient (ICC) was 0.25, indicating that 25% of the total variance in cortical bone thickness was attributable to differences between patients, while 75% reflected variability between measurement points within the same patient.

### 3.5. Limitations

This study has several limitations that should be acknowledged. First, the retrospective design restricts control over patient selection and clinical variables, and causal relationships cannot be inferred. Second, although the sample size was relatively large, some subgroups (e.g., patients with thyroid disease) were underrepresented, which may limit the generalizability of the findings. Third, measurements were performed using a single CBCT system and software, which ensures consistency but may restrict comparability with studies employing different imaging protocols. Additionally, no statistically significant associations were detected between cortical bone thickness and smoking, diabetes, or thyroid disease in this sample. Given the small and uneven sub-group sizes, these results should not be interpreted as evidence of absence of an effect, but rather as a limitation of statistical power. Finally, anatomical variations and differences in clinical conditions across populations were not fully accounted for, suggesting the need for prospective, multicenter studies with larger and more diverse cohorts to validate these results.

## 4. Discussion

The present study demonstrated significant variation in cortical bone thickness between anatomical measurement points (*p* < 0.001). The thinnest bone was observed at point B (1.16 ± 0.30 mm), while the thickest values were recorded at points A (1.38 ± 0.51 mm) and F (1.37 ± 0.46 mm). The linear mixed model confirmed a statistically significant influence of sex, with men exhibiting thinner cortical bone compared to women (difference = 0.098 mm, *p* = 0.048), and of age, with bone thickness decreasing by approximately 0.005 mm per year (*p* = 0.010). In contrast, health-related factors such as smoking, diabetes, and thyroid disease showed no significant associations with cortical thickness.

The concept of anchoring implants in the second cortical bone layer was first introduced by D. Gabraccio in the late 1970s using one-piece bicortical screws, a technique that gained popularity primarily in Italy [15]. In the context of two-piece implants, Brånemark and colleagues subsequently described second cortical anchorage in the maxilla in 1984 as a treatment option for cases of advanced bone atrophy [16]. This approach required perforation of the inner cortical plate so that the load-bearing implant threads engaged with the denser bone, often resulting in implant protrusion into the nasal cavity or maxillary sinus [16]. In the mandible, second cortical anchorage was first reported by Kermakov [17], who utilized the mylohyoid buttress. However, these early approaches were limited by the implant lengths required to reach the second cortical and by the prosthetic solutions available at the time.

In contemporary implantology, reconstruction of the edentulous maxilla frequently employs second cortical anchorage through concepts such as all-on-X, nasalis implants, strategic bicortical implants, or even subperiosteal implants [18,19,20]. These approaches take advantage of the cortical bone of the nasal floor and lateral walls, enabling high insertion torque (IT) values, often reaching 80–90 Ncm. The literature suggests that a cortical thickness of >1.5 mm is preferred for optimal stability and long-term success, with a minimum of 1.0 mm required for predictable anchorage [21]. However, these thresholds have been primarily established for the alveolar crest and buccolingual cortical walls. Further studies are therefore needed to assess the clinical relevance of nasal and second cortical bone thickness for long-term implant success, particularly under immediate loading protocols.

Our findings support the use of nasal floor and lateral wall cortices as alternative anchorage sites in implantology. Specifically, points A and F demonstrated mean cortical thicknesses >1.35 mm, approaching or exceeding the threshold considered favorable for predictable bicortical engagement (>1.0 mm minimum, ideally >1.5 mm). These POI regions may therefore provide enhanced primary implant stability, particularly in immediate loading protocols where insertion torque values of 35–45 Ncm are desired. In contrast, thinner sites (e.g., point B) should be approached with caution when planning for bicortical anchorage.

The present findings align with previous biomechanical and clinical studies that have established a minimum cortical thickness threshold for predictable implant anchorage. Finite-element simulations and insertion-torque studies have shown that dental implants require a cortical layer of at least 1.0 mm, and preferably around 1.5 mm, to obtain strong primary stability and an even distribution of mechanical stress during functional loading. [21]. Motoyoshi et al. reported that optimal tightening torque for orthodontic mini-implants correlated with cortical bone thickness above 1.0 mm, while Di Stefano et al. confirmed similar thresholds for dental implants in maxillary bone [2,21]. In this context, the median values observed at points A (1.36 mm) and F (1.35 mm) approach or exceed these stability benchmarks, suggesting that these regions may provide sufficient cortical engagement to support immediate loading protocols or bicortical fixation strategies. Conversely, thinner regions such as point B (≈1.15 mm) may be less favorable for achieving the required insertion torque, especially in cases of low trabecular density or reduced bone quality. These comparisons reinforce the clinical applicability of our anatomical mapping for pre-surgical planning in atrophic maxillae.

While we did not detect statistically significant associations with smoking, diabetes, or thyroid disease, the limited subgroup sizes prevent definitive conclusions. Larger prospective studies are required to evaluate systemic risk factors in relation to cortical bone thickness.

In cases of severe maxillary atrophy, the resorption of the alveolar process significantly reduces available bone for conventional implant anchorage. Under such conditions, alternative strategies including bicortical engagement of the nasal floor or lateral wall have been explored. Although these approaches are restricted to specific clinical scenarios, they can provide mechanical stability when alveolar support is insufficient. Early reports by Gabraccio, Brånemark, and others introduced these concepts, but anatomical and prosthetic limitations constrained their wider adoption [15,16]. Our study follows up on these observations by providing a quantitative evaluation of cortical thickness in the nasal floor and lateral wall to assess their potential for strategic implant anchorage. Overall, the results highlight that precise anatomical assessment of nasal cortical bone thickness can serve as a practical guide for selecting implant sites and trajectories that maximize primary stability.

## 5. Conclusions

The awareness of cortical bone thickness in anatomical structures such as the floor of the nose and its lateral wall is of great importance for implant planning and placement. CBCT imaging allows precise assessment of available cortical bone, enabling clinicians to adapt techniques and implant positioning to enhance patient safety and improve clinical outcomes.

This study demonstrated that measurement location is the key determinant of bone thickness within the nasal floor region. Sites adjacent to the lateral wall and the anterior nasal spine–nasal septum region exhibited significantly greater cortical thickness compared to other measurement points, underscoring their potential for reliable implant anchorage.

With respect to demographic influences, men showed statistically thinner bone compared to women (mean difference: 0.098 mm); however, this difference is unlikely to be clinically relevant. Health-related factors, including smoking, diabetes, and thyroid disease, had no significant impact on cortical bone thickness. Overall, anatomical location emerged as the most important predictor of cortical bone thickness, outweighing demographic and systemic health variables. These findings provide important reference data for risk assessment and surgical planning in implant dentistry.

### Recommendations

Clinical practice: Anatomical location should be prioritized when assessing cortical bone thickness for implant anchorage in the nasal region.

Further research: Prospective, multicenter studies with larger patient cohorts are required to confirm these findings and to explore additional influencing factors.

Diagnostic protocols: Standardization of measurement points is recommended to enhance reproducibility and allow for meaningful comparison across studies.

## Figures and Tables

**Table 2:** Results of the linear mixed-effects model evaluating the influence of risk factors on cortical bone thickness.

Variable	Coefficient (β)	Standard Error	*p*-Value	95% CI
Sex (male)	−0.098	0.050	0.048	[−0.196, −0.001]
Smoking	0.024	0.062	0.700	[−0.097, 0.144]
Diabetes	0.118	0.082	0.152	[−0.043, 0.280]
Thyroid disorders	0.055	0.091	0.546	[−0.123, 0.233]
Age (centered)	−0.005	0.002	0.010	[−0.008, −0.001]

**Table 3:** Variance structure of the linear mixed-effects model for cortical bone thickness.

Component	Variance	Proportion of Total Variance
Between patients	0.055	25%
Within patients (residual)	0.166	75%
Total variance	0.221	100%

## Data Availability

The data presented in this study are available on reasonable request, after the signature of a formal data sharing agreement in anonymous form, from the corresponding author because they are protected by privacy.
